# Monitoring of Ebola Virus Makona Evolution through Establishment of Advanced Genomic Capability in Liberia

**DOI:** 10.3201/eid2107.150522

**Published:** 2015-07

**Authors:** Jeffrey R. Kugelman, Michael R. Wiley, Suzanne Mate, Jason T. Ladner, Brett Beitzel, Lawrence Fakoli, Fahn Taweh, Karla Prieto, Joseph W. Diclaro, Timothy Minogue, Randal J. Schoepp, Kurt E. Schaecher, James Pettitt, Stacey Bateman, Joseph Fair, Jens H. Kuhn, Lisa Hensley, Daniel J. Park, Pardis C. Sabeti, Mariano Sanchez-Lockhart, Fatorma K. Bolay, Gustavo Palacios, on behalf of US Army Medical Research Institute of Infectious Diseases, National Institutes of Health, Integrated Research Facility–Frederick Ebola Response Team 2014–2015

**Affiliations:** US Army Medical Research Institute of Infectious Diseases, Fort Detrick, Frederick, Maryland, USA (J.R. Kugelman, M.R. Wiley, S. Mate, J.T. Ladner, B. Beitzel, K. Prieto, T. Minogue, R.J. Schoepp, K.E. Schaecher, S. Bateman, M. Sanchez-Lockhart, G. Palacios);; Liberian Institute for Biomedical Research, Charlesville, Liberia (L. Fakoli, F. Taweh, F.K. Bolay);; Naval Medical Research Unit 3, Cairo, Egypt (J.W. Diclaro);; Integrated Research Facility at Fort Detrick, National Institute of Allergy and Infectious Diseases, National Institutes of Health, Frederick (J. Pettitt, J.H. Kuhn, L. Hensley);; Foundation Merieux, Washington, DC, USA (J. Fair);; Broad Institute, Cambridge, Massachusetts, USA (D.J. Park, P.C. Sabeti);; Center for Systems Biology, Harvard University, Cambridge (P.C. Sabeti)

**Keywords:** viral countermeasures, genomics, viral hemorrhagic fever, Ebola virus, filovirus, negative-strand RNA virus, viruses, Liberia

## Abstract

To support Liberia’s response to the ongoing Ebola virus (EBOV) disease epidemic in Western Africa, we established in-country advanced genomic capabilities to monitor EBOV evolution. Twenty-five EBOV genomes were sequenced at the Liberian Institute for Biomedical Research, which provided an in-depth view of EBOV diversity in Liberia during September 2014–February 2015. These sequences were consistent with a single virus introduction to Liberia; however, shared ancestry with isolates from Mali indicated at least 1 additional instance of movement into or out of Liberia. The pace of change is generally consistent with previous estimates of mutation rate. We observed 23 nonsynonymous mutations and 1 nonsense mutation. Six of these changes are within known binding sites for sequence-based EBOV medical countermeasures; however, the diagnostic and therapeutic impact of EBOV evolution within Liberia appears to be low.

## Abstract 2

The effects of EBOV evolution on diagnostic assays and therapeutic drugs appear to be low.

The outbreak of Ebola virus disease (EVD) in Western Africa that started in November 2013 (*1*) is the largest recorded filovirus disease outbreak. As the outbreak continues, public health and emerging infectious disease officials have declared a continuing need for real-time monitoring of Ebola virus (EBOV) evolution (*2*,*3*). As of March 11, 2015, a total of 41% of reported cases had been fatal (*4*). By the end of March 2015, the intensity of the outbreak, which throughout its course affected 6 Western Africa countries, appeared to be receding, with near 0 activity in Liberia and no cases in Mali, Nigeria, and Senegal. However, EBOV continues to spread in Guinea and Sierra Leone. The epidemic is still causing more infections per week than have been recorded in previous EVD outbreaks (*5*). Therefore, public health officials continue to use media to maintain public awareness, to advocate for diligent handwashing and use of other protective measures, and to avoid complacency that could lead to reemergence (*5*). Vigilance is of paramount importance because currently used assays for EVD diagnosis, and many medical countermeasures in development, were designed using EBOV reference genome variants from previous outbreaks (*6*–*9*). Therefore, monitoring EBOV genomic drift is crucial because genetic changes can affect the efficacy of sequence-based therapeutics and diagnostics.

The size and spread of the current EVD outbreak reinforces the need to build public health infrastructure, including state-of-the-art diagnostic and surveillance capabilities, to implement and maintain effective EVD monitoring, treatment, and prevention platforms. The Liberian Institute for Biomedical Research (LIBR), established in 1975, is located in Charlesville, 50 km southeast of Liberia’s capital, Monrovia. As of April 2, 2015, it is one of the few local facilities within Liberia processing clinical samples from persons suspected to have EVD. A consortium comprising US government and nongovernment agencies has been working with the Liberian government to equip LIBR with advanced genomic sequencing capabilities. These capabilities are dedicated primarily to EVD surveillance activities, including genome sequencing of EBOV-positive samples. The new LIBR Genome Center has a Miseq sequencer (Illumina, San Diego, CA, USA) and ancillary supporting capabilities, including electrophoresis for qualification, fluorometry for quantitation, PCR for amplification, and fully functional computational analysis capabilities to perform pathogen discovery and microbial genome characterization. The US Army Medical Research Institute of Infectious Diseases (USAMRIID) Center for Genome Sciences supports LIBR operation and development. Sample preparation procedures under biosafety containment are provided within the same building complex by the Liberian National Reference Laboratories, operated by USAMRIID and the National Institutes of Health Integrated Research Facility Ebola Response Team (Fort Detrick, Frederick, MD, USA). Throughput at the LIBR Genome Center is 10–20 samples (≈10 billion bases of sequence data) per week, with a target turnaround time of 7 days from sample receipt for high-priority samples. To ensure long-term sustainment of surveillance-based sequencing capabilities, local biomedical scientists have been trained and can proficiently perform all daily activities.

Here we demonstrate the utility and capabilities of the LIBR Genome Center. With the immediate goal of continuing the natural history characterization of the EBOV Makona variant (EBOV/Mak [*10*]) currently circulating in Western Africa and to support ongoing clinical trials to evaluate candidate medical countermeasures, we describe 25 EBOV genome sequences from the first 5 sequencing runs conducted at the LIBR Genome Center. We chose these samples for full-genome characterization from ≈1,700 available samples on the basis of high viral load (cycle threshold [C_t_]) value <24) and date of collection to ensure up-to-date temporal coverage.

## Materials and Methods

### Samples

**Figure 1:**
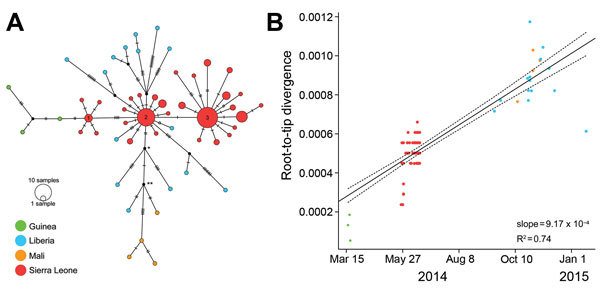
A) Median-joining haplotype network constructed from a full-genome alignment of 122 clinical Ebola virus Makona (EBOV/Mak) isolates (list of isolates in Technical Appendix 3). Each colored vertex represents a sampled viral haplotype, with the numbered vertices representing the centers of the 3 clusters described in (*12*). All sampled isolates from Liberia originated from cluster 2. The size of each vertex is relative to the number of sampled isolates, and the colors indicate country of origin. Hatch marks indicate the number of mutations along each edge. Because of missing data, 2,764 sites (14.6% of total genome) were excluded from the analysis, including 26 sites with variability among isolates (16.7% of all variable sites). B) Root-to-tip distance correlates well with test date and estimates a rate of evolution equal to 9.17 × 10^−4^ substitutions/site/year. This analysis comprises 110 clinical EBOV/Mak isolates collected during March 17, 2014–January 20, 2015 (Technical Appendix 3, isolates with dates).

We chose samples from 25 patients from the larger collection (≈1,700 positive cases) on the basis of diagnostic C_t_ values that indicated a high enough viral load to provide a full genome (C_t_<24), beginning with the most recent available at the time of preparation in February 2015. Sampling continued with progressively older samples to describe the lineages most likely to still be circulating at the time. These patients were treated in 7 different Ebola treatment units and had resided in 7 of the 15 counties in Liberia (Table 1; Technical Appendix 1 Figure 1). Plasma or oral swab samples from which viral RNA was recovered and sequenced were tested at LIBR during September 23, 2014–February 14, 2015. Patients’ ages were as follows: 1 infant (1 year), 6 children (2–15 years), 8 young adults (18–35 years), and 10 middle-aged adults (42–67 years). The male:female ratio was 2:1. However, among ≈1,700 samples at LIBR from persons with EVD, the ratio was close to 1:1 (48%/52%), and viral load did not differ by patient sex, which demonstrates that our higher ratio is a sampling artifact.

**Table 1:** Characteristics of Ebola virus samples from selected patients, Liberia, September 2014–February 2015*

Sample ID	Patient age, y/sex	County of residence	Test date	Sample type	Average C_t_ value†
LIBR10054	53/M	Bomi	2014 Sep 23	Plasma	20.5
LIBR10053	42/NA	Not Available	2014 Oct 1	NA	22
LIBR0058	67/M	Rivercess	2014 Nov 5	NA	22
LIBR0059	27/M	Rivercess	2014 Nov 5	NA	22
LIBR0073	27/M	Grand Bassa	2014 Nov 6	Plasma	18.5
LIBR0067	29/NA	Bomi	2014 Nov 6	Plasma	21
LIBR0063	3/F	Montesserrado	2014 Nov 6	Oral swab	17.5
LIBR0093	47/M	Montesserrado	2014 Nov 6	Plasma	15.5
LIBR0092	18/F	Montesserrado	2014 Nov 8	Plasma	21
LIBR0090	62/F	Margibi	2014 Nov 8	Plasma	22
LIBR0116	4/F	Grand Bassa	2014 Nov 10	Plasma	19
LIBR0168	15/M	Bomi	2014 Nov 13	Plasma	22.5
LIBR0176	42/M	Montesserrado	2014 Nov 14	Oral swab	22.5
LIBR0173	64/M	Montesserrado	2014 Nov 14	Oral swab	22
LIBR0286	9/F	Grand Cape Mount	2014 Nov 22	Plasma	22
LIBR0333	35/F	Grand Cape Mount	2014 Nov 25	Plasma	19.5
LIBR0423	45/F	Montesserrado	2014 Dec 3	Plasma	21.5
LIBR0430	1/M	Grand Bassa	2014 Dec 3	Oral swab	23.5
LIBR0503	8/F	Sinoe	2014 Dec 10	Plasma	23
LIBR0505	29/F	Sinoe	2014 Dec 10	Plasma	25
LIBR0605	2/M	Montesserrado	2014 Dec 20	Oral swab	23
LIBR0624	53/M	Montesserrado	2014 Dec 22	Plasma	19.5
LIBR0993	33/M	Montesserrado	2015 Jan 20	Plasma	19.5
LIBR1195	35/M	Margibi	2015 Feb 2	Oral swab	22.5
LIBR1413	56 M	Montesserrado	2015 Feb 14	Plasma	22.5

*C_t_, cycle threshold; ID, identification; NA, not available. †C_t_ values used as indicator of viral load obtained from 2 diagnostic assays performed on all samples (Kulesh-TM and Kulesh-MGB [*9*]).

### Sample Processing

RNA was converted to cDNA and amplified by using sequence-independent single-primer amplification (*11*). Amplified cDNA was quantified with a Qubit 3.0 fluorometer (Life Technologies, Carlsbad, CA, USA) and used as the starting material for the Illumina Nextera XT DNA library preparation kit (Illumina). Sequencing was performed on an Illumina Miseq by using either V2 or V3 reagent kits (Illumina) with a minimum of 2 × 151 cycles per run.

### Genome Assembly

We assembled EBOV genomes by aligning reads to the genome of Ebola virus/H.sapiens-wt/SLE/2014/Makona-G3686.1 (GenBank accession no. KM034562.1) (*12*). Amplification primers were removed from the sequencing reads by using Cutadapt version 1.21 (*13*), and low-quality reads/bases were filtered by using Prinseq-lite version 0.20.4 (-min_qual_mean 25 -trim_left 20 -min_len 50) (*14*). Reads were aligned to the reference genome by using DNAStar Lasergene nGen (DNAStar, Madison, WI, USA), and a new consensus was generated by using a combination of Samtools v0.1.18 (*15*) and custom scripts. Only bases with Phred quality score >20 were used in consensus calling, and a minimum of 3× read-depth coverage, in support of the consensus, was required to make a call; positions lacking this depth of coverage were treated as missing (i.e., called as “N”).

### Genetic Analysis

Consensus sequences generated here were aligned with additional publically available EBOV genomes by using Sequencher version 5.2.3 (Gene Codes, Ann Arbor, MI, USA). SnpEff version 4.1b (build 2015-02-13) was used to annotate all single-nucleotide polymorphisms (SNPs) by using the genome of Ebola virus/H.sapiens-wt/GIN/2014/Makona-C15 (GenBank accession no. KJ660346.2) as a reference (*16*). All 25 genomes from Liberia were used to identify variable sites. For the rest of the genetic analysis, we used only the 14 sequences with >90% genome coverage. A median-joining haplotype network was constructed in PopART version 1.7.2 (http://popart.otago.ac.nz). Path-O-Gen version 1.4 (*17*) was used to calculate the root-to-tip distances by using a maximum-likelihood phylogeny (PhyML version 3.0 (*18*); general time reversible model) with rooting based on the EBOV phylogeny published by Gire et al. (*12*). BEAST version 1.8.2 (*17*) was used to estimate the mutation rate and the time to the most recent common ancestor for several evolutionary lineages that included Liberia EBOV isolates. For analysis, we divided the alignment into 3 partitions (i.e., first + second codon sites, third codon site, and noncoding sites). The substitution process was modeled independently for each by using the Hasegawa, Kishino, and Yano model with 4 gamma categories. An exponential growth coalescent model was used with a strict clock. The XML input file is available on request from the authors.

## Results

From the first 5 sequencing runs, we obtained 25 EBOV genomes with >50% coverage; 6 of these were coding complete (Table 2) (*19*). These genomes contained 97 new sequence variants: 47 synonymous, 23 nonsynonymous, 1 nonsense, and 26 noncoding mutations (Technical Appendix 2). Multiple distinct evolutionary lineages were detected, but all were consistent with a single introduction of a cluster 2–type (*12*) virus into Liberia followed by within-country diversification (Figure 1, panel A). Because 19 of the 25 genomes had calls at all 5 positions that discriminate clusters 1, 2, and 3, we have high confidence in cluster attribution. 

**Table 2:** Next-generation sequencing of 25 Ebola virus isolates derived from selected patients sampled, Liberia, September 2014–February 2015

Sample ID	Coverage, %*	No. reads	Finishing category†	GenBank accession no.
LIBR0093	99.4	169,000	Coding complete	KR006947
LIBR0116	97.9	710,168	Coding complete	KR006948
LIBR10054	98	2,150,725	Coding complete	KR006964
LIBR0073	98.5	3,351,831	Coding complete	KR006944
LIBR0503	98.9	3,193,168	Coding complete	KR006956
LIBR0286	98.3	1,731,953	Coding complete	KR006952
LIBR0993	96.5	750,000	Standard draft	KR006960
LIBR0423	97.1	2,676,454	Standard draft	KR006954
LIBR0333	97.1	1,775,653	Standard draft	KR006953
LIBR10053	98	1,691,652	Standard draft	KR006963
LIBR0067	97	2,403,590	Standard draft	KR006943
LIBR0092	93.9	2,758,142	Standard draft	KR006946
LIBR0090	93.1	1,422,271	Standard draft	KR006945
LIBR1413	88.2	2,500,000	Standard draft	KR006962
LIBR0058	91.4	1,632,978	Standard draft	KR006940
LIBR0176	89.4	1,907,863	Standard draft	KR006951
LIBR0168	89.2	1,221,075	Standard draft	KR006949
LIBR0505	83.8	741,165	Standard draft	KR006957
LIBR1195	73.1	2,200,773	Standard draft	KR006961
LIBR0624	68	1,550,511	Standard draft	KR006959
LIBR0063	69	2,883,384	Standard draft	KR006942
LIBR0173	72.3	1,456,490	Standard draft	KR006950
LIBR0059	59.1	851,606	Standard draft	KR006941
LIBR0605	64.7	1,587,732	Standard draft	KR006958
LIBR0430	56.2	3,139,009	Standard draft	KR006955

*Percentage of genome bases (of 18,959 total bases) called in the consensus sequences (requires >3× coverage with base quality >20). †Categories are defined in (*19*).

Molecular dating places the common ancestor to all of the sampled isolates from Liberia during May 2–July 9, 2014 (95% highest posterior density [HPD] interval), which corresponds with the early days of the outbreak in Monrovia (*3*). However, we cannot rule out ongoing EBOV exchange among EVD-infected countries. In fact, shared ancestry among 3 isolates from Liberia and the 4 available sequences from Mali suggests some level of international movement. We estimated dates associated with 2 nodes along the shared Liberia/Mali EBOV lineage (labeled * and ** in Figure 1, panel A**)**; these estimates ranged from July 6 through September 15, 2014, and from July 26 through September 27, 2014, respectively (95% HPD). Overall, collection dates correlated well with root-to-tip distances within the Western Africa EVD outbreak (Figure 1, panel B). Linear regression analysis (using the lm function in R version 3.1.1; http://www.r-project.org/) estimated an overall rate of change of 9.17 × 10^−4^ substitutions/site/year (± 5.23 × 10^−5^). Bayesian analysis estimated a similar rate of change of 9.44–15.67 × 10^−4^ substitutions/site/year (95% HPD).

We reviewed all publicly available genomic information for EBOV/Mak (122 genome sequences [*1*,*12*]) to evaluate the effect of genomic drift on biomedical countermeasures (drugs and diagnostic assays). We assessed the potential impact of intra-outbreak genetic divergence on 13 drugs and 2 diagnostic assays (known to be used in Liberia) with the same approach previously used (*6*). Two sequence-binding treatment modalities are available for postexposure treatment of EVD: small interfering RNAs (siRNAs) (*20*) and phosphorodiamidate morpholino oligomers (*21*) targeting *L*, *VP24*, and/or *VP35* gene transcripts, and passive immunotherapy based on antibodies or antibody cocktails targeting EBOV glycoprotein (*22*–*26*). These treatments inhibit viral replication by targeting viral transcripts for degradation (siRNA) or by blocking translation (phosphorodiamidate morpholino oligomers), or they acutely neutralize the virus to enable the host to mount an effective immune response (passive immunotherapy). These countermeasures were originally designed specifically against sequences obtained during previous outbreaks (*20*,*27*) or were generated against their glycoproteins (e.g., the monoclonal antibodies [mAbs] were obtained after immunization with Ebola virus/H.sapiens-tc/COD/1995/Kikwit-9510621 [EBOV/Kik-9510621] [*28*]).

**Figure 2:**
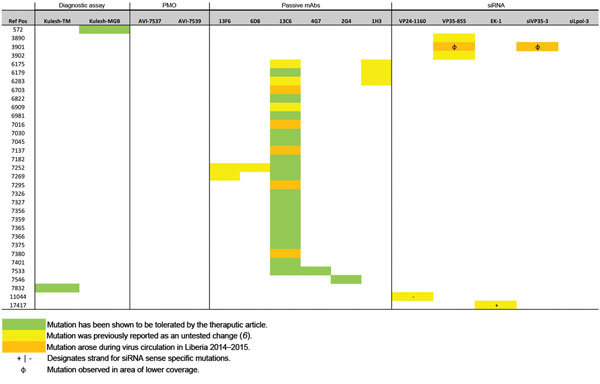
Mutation analysis of candidate therapeutic drug and diagnostic binding sites used in outbreak of Ebola virus (EBOV) disease, Western Africa. A single-nucleotide polymorphism (SNP) table is combined with a heat map based on 2 categories: 1) mutations tolerated by the therapeutic drug or diagnostic target (highlighted in green); 2) mutations within the binding region of a therapeutic drug or diagnostic assay that have not yet been tested (highlighted in yellow/orange) (*20**–**24**,**27**,**30**,**31*). Changes previously described are highlighted in yellow; changes that appeared during circulation in Liberia are highlighted in orange. The reference nucleotide positions reported here are in relation to EBOV/Kik-9510621 (GenBank accession no. AY354458), which is one of the primary isolates used as reference for developing these therapeutic drugs and diagnostic assays. A summary of the changes to the probes is available in Technical Appendix 1 Table. PMO, phosphorodiaminate morpholino oligomer, mAB, monoclonal antibody; siRNA, small interfering RNA; Ref pos, reference positive; VP, viral protein.

Since the Western Africa outbreak began, at least 33 viral mutations have occurred that could affect countermeasures. We previously reported 27 of these mutations (*6*). Twenty-six (79%) mutations induced nonsynonymous changes to epitopes recognized by mAbs included in passive immunotherapy cocktails. Another 5 (15%) were located in published binding regions of siRNA-based therapeutic drugs. Tekmira has adjusted its siRNAs to account for 4 of these 5 changes since its initial publication (*29*; E.P. Thi et al., unpub. data). The final 2 mutations were located in the published binding region of primers or probes for quantitative PCR diagnostic tests that have been used during outbreak control activities in Liberia: 1 change each in the binding sites of the Kulesh-TM assay and the Kulesh-MGB assay (*9*). Nevertheless, reassessment of the assays at USAMRIID has suggested that the changes will be tolerated without loss in sensitivity (data not shown). Changes in all EBOV/Mak sequences are considered “interoutbreak” (n = 23); changes observed only in some sequences from Western Africa are considered “intraoutbreak” sites (n = 10, EBOV-WA <100%). We also examined the binding sites of an additional 18 publicly available EBOV quantitative PCRs, which might (or might not) also be used in Western Africa (Technical Appendix 1 Figure 2, Technical Appendix 1 Table). We observed 25 changes, of which 6 were reported previously (*12*). Each SNP has the potential to affect the efficacy of available therapeutic drugs (original and updated versions) or diagnostic assays (Table 3; Figure 2; Technical Appendix 1 Figure 2, Technical Appendix 1 Table; nucleotide positions are reported relative to EBOV/Kik-9510621, for consistency [*6*]).

**Table 3:** Mutation analysis of candidate therapeutic drug and diagnostic binding sites for EBOV*

Reference position	Type	Reference base	Called base	EBOV-WA, %	EBOV-LIB, %	Codon	Feature name
850	SNP	A	G	100	100	G:GGA @ 127 → G:GGg	NP
852	SNP	A	G	100	100	K:AAA @ 128 → R:AgA	NP
895	SNP	A	G	100	100	T:ACA @ 142 → T:ACg	NP
907	SNP	T	C	1	0	N:AAT @ 146 → N:AAc	NP
919	SNP	T	C	100	100	F:TTT @ 150 → F:TTc	NP
1288	SNP	A	T	1	0	V:GTA @ 273 → V:GTt	NP
1495	SNP	A	G	100	100	Q:CAA @ 342 → Q:CAg	NP
1498	SNP	C	T	1	4	L:CTC @ 343 → L:CTt	NP
1507	SNP	T	A	100	100	A:GCT @ 346 → A:GCa	NP
1552	SNP	C	T	100	100	R:CGC @ 361 → R:CGt	NP
1862	SNP	A	G	100	100	S:AGC @ 465 → G:gGC	NP
6359	SNP	T	C	100	100	N:AAT @ 107 → N:AAc	GP
6909	SNP	T	A	1	0	W:TGG @ 291 → R:aGG	GP
7730	SNP	G	A	100	100	E:GAG @ 564 → E:GAa	GP
7775	SNP	A	G	100	100	L:CTA @ 579 → L:CTg	GP
7778	SNP	C	A	100	100	R:CGC @ 580 → R:CGa	GP
10252	SNP	A	T	1	4		
10253	SNP	A	G	1	0		
12694	SNP	T	A	100	100	I:ATT @ 371 → I:ATa	L
12886	SNP	A	C	2	0	L:CTA @ 435 → L:CTc	L
12952	SNP	A	G	100	100	L:CTA @ 457 → L:CTg	L
13267	SNP	C	T	100	100	T:ACC @ 562 → T:ACt	L
13607	SNP	G	A	1	4	V:GTC @ 676 → I:aTC	L
13624	SNP	T	G	1	0	N:AAT @ 681 → K:AAg	L
13630	SNP	A	G	100	100	P:CCA @ 683 → P:CCg	L

*EBOV, Ebola virus; GP; glycoprotein, ; L, RNA-dependent RNA polymerase; LIB, Liberia; NP; nucleoprotein; SNP, single-nucleotide polymorphism; WA, Western Africa.

Several of the 27 previously identified changes (green in Figure 2) already have been demonstrated to be tolerated while maintaining efficacy (*24*,*30*,*32*–*34*), thus minimizing their potential effect (*6*). Six of these 33 SNPs (EBOV-LIB <100%; orange in Figure 2) appeared during the surveillance period of this study (September 23, 2014–February 14, 2015) in samples obtained in Liberia (*12*). None of these changes have been previously associated with EBOV resistance to any therapeutic drug. Five of the new changes might affect 1 of the components of the ZMapp antibody cocktail (mAb 13C6). However, the conformational target site for this antibody (positions 1–295, soluble glycoprotein) is broader in length and more poorly defined than the other sequence-based countermeasure targets considered in our risk assessment. The sixth mutation might affect the binding site of the siRNA viral protein (VP) 35 target (for that particular sample, the mutation appears in an area of low sequencing coverage depth). Thus, when these new changes are combined with the changes observed previously (yellow in Figure 2), we can conclude that retesting several therapeutic drugs against isolates currently circulating might be necessary to determine whether any of these mutations impact their efficacy. In particular, it is important to reevaluate drugs that include mAb 13C6 (part of the ZMapp, ZMAb, and MB-003 antibody cocktails), mAb 13F6 (part of MB-003), mAb 1H3 (part of ZMAb), and the siRNA VP35 targets (Table 3, Figure 2) (*6*).

## Discussion

Our study details the establishment of a genomic sequencing and analysis center within Liberia for real-time monitoring of viral evolution. The initial sequences generated at this facility have provided a first glimpse into EBOV/Mak evolution from the end of 2014 to the beginning of 2015. Although genetically diverse, the viruses circulating in Liberia during this period are consistent with a single introduction event followed by diversification within Liberia. The cluster 2 haplotype from which all the sampled Liberia sequences radiate is thought to have been circulating in Guinea and Sierra Leone during late May 2014 (*12*). Moreover, it was the second most common sequence detected in Sierra Leone during late May through mid-June (*12*). Introduction of this haplotype from either of these neighboring countries could have resulted in the sampled diversity; however, we cannot rule out the possibility of multiple introductions. Additional spatial and temporal sampling within Liberia, Guinea, and Sierra Leone will help to differentiate these 2 scenarios.

The 25 Liberia EBOV/Mak genomes included 23 nonsynonymous mutations and 1 nonsense mutation that have not previously been seen in Western Africa (although some of these mutations have been observed in EBOV isolates from previous EVD outbreaks). A nonsense mutation, which is present within 2 of the 25 sequences, is predicted to result in premature truncation (6 aa) of VP30. VP30 is an essential protein for viral transcription; it is needed for the RNA-dependent RNA polymerase (L) to read beyond a *cis*-RNA element in the nucleoprotein mRNA 5′ untranslated region (*35*) and is required to reinitiate transcription at gene junctions (*36*). Moreover, VP30 phosphorylation modulates the composition and function of the RNA synthesis machinery (*37*). To our knowledge, no functional domains have been described in the truncated region. Further characterization is needed to determine whether this or any of the other detected mutations impacted the relative fitness of the affected EBOV isolates. Within Liberia, geography showed little correlation with phylogeny; most EBOV lineages within Liberia appear to be geographically widespread within the sampled regions.

Previous analysis of EBOV/Mak genomes from Sierra Leone and Guinea suggests that the evolutionary rate within the current EVD outbreak might be higher than the rate between outbreaks (*12*). After incorporation of sequences from Liberia, which were collected later in the outbreak, our estimates of substitution rate fell between the previous estimates for EBOV/Mak only and for all EBOV (*12*,*38*). As more sequence data become available, it will be interesting to see whether a significant change in the evolutionary rate can be detected within the current EVD outbreak.

Our ability to quantify international EBOV exchange is limited because few isolates from other countries were available during the sampled timeframe. However, shared ancestry between isolates from Mali and 3 isolates from Liberia suggests at least 1 transmission event across national borders (*3*). All EVD cases in Mali have been attributed to movement of infected persons into Mali from Guinea (*39*). With the current dataset, it is impossible to say whether the shared Liberia/Mali lineage originated in Liberia and was then transported to Mali through Guinea or whether the lineage emerged in Guinea and later moved independently to Liberia and Mali. Active EBOV outbreaks were occurring in both Liberia and Guinea during the period estimated for the emergence of this shared lineage (July–September 2014).

The genomic changes observed for EBOV/Mak during its circulation in Liberia append 5 additional mutations to the list of changes that might affect the binding of the 13C6 mAb, a component of ZMapp. All of these changes, however, were present at relatively low frequency (<12%) in our current sample, and none of the sampled lineages have accumulated >1 change per therapeutic drug type. We observed no significant changes (i.e., likely to affect efficacy) in the binding sites for the 2 diagnostic assays known to be used in Liberia. Overall, no dramatic changes were observed in the samples evaluated; the risk assessment for the impact of genomic drift during the outbreak should remain low. As previously stated (*6*), our analysis is not without caveats. Our current analysis covers only the late period of the outbreak in Liberia; no analysis has yet been published with data for similar time points from Guinea or Sierra Leone. In addition, to complete our assessment of the evolution of EBOV in Liberia, an earlier period of time from the introduction of the virus in March 2014 to early September 2014 needs to be investigated.

Our findings offer a concise evaluation of the potential impact of the evolution of EBOV/Mak based on genome reconstruction of 25 isolates from Liberia obtained during September 2014–February 2015. This work would not have been possible without the establishment of a genomic surveillance capability in Liberia, which emphasizes the need for global sequencing capabilities to be part of the first response during future virus outbreaks.

Technical Appendix 1 Map of Liberia counties showing the 25 Ebola virus (EBOV) isolates described in this study; mutation analysis of diagnostic binding sites; and diagnostic probe information.

Technical Appendix 2 Consensus-level variants in 25 Liberian Ebola virus Makona genomes relative to reference genome Ebola virus/H.sapiens-wt/GIN/2014/Makona-C15.

Technical Appendix 3 Ebola virus Makona isolates used in genetic analyses.
