# Supplementary material for: Monitoring of Ebola Virus Makona Evolution through Establishment of Advanced Genomic Capability in Liberia
Source: Emerg Infect Dis. 2015 Jul;21(7):1135–43. doi: 10.3201/eid2107.150522 (PMC4816332; doi:10.3201/eid2107.150522)
Supplement: Technical Appendix 1 — Map of Liberia counties showing the 25 Ebola virus (EBOV) isolates described in this study; mutation analysis of diagnostic binding sites; and diagnostic probe information. [file 15-0522-Techapp-s1.pdf]

Article DOI: http://dx.doi.org/10.3201/eid2107.150522

Monitoring of Ebola Virus Makona Evolution through Establishment of Advanced Genomic Capability in Liberia

Technical Appendix 1

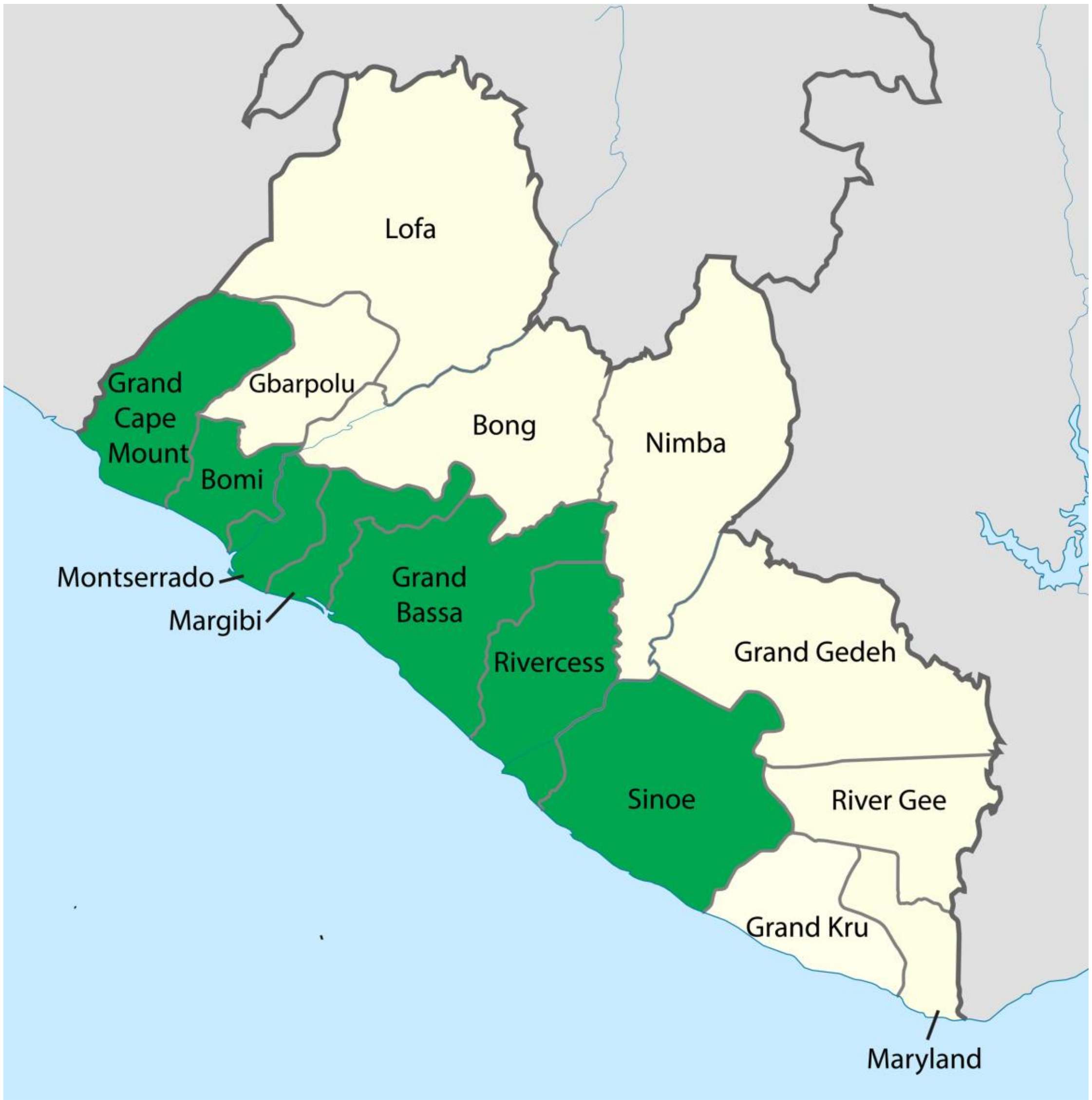

Technical Appendix 1 Figure 1. Map of Liberia counties showing the 25 Ebola virus (EBOV) isolates described in this study. Samples were collected from persons in 7 coastal Liberian counties (highlighted in green) during September 2014–February 2015.

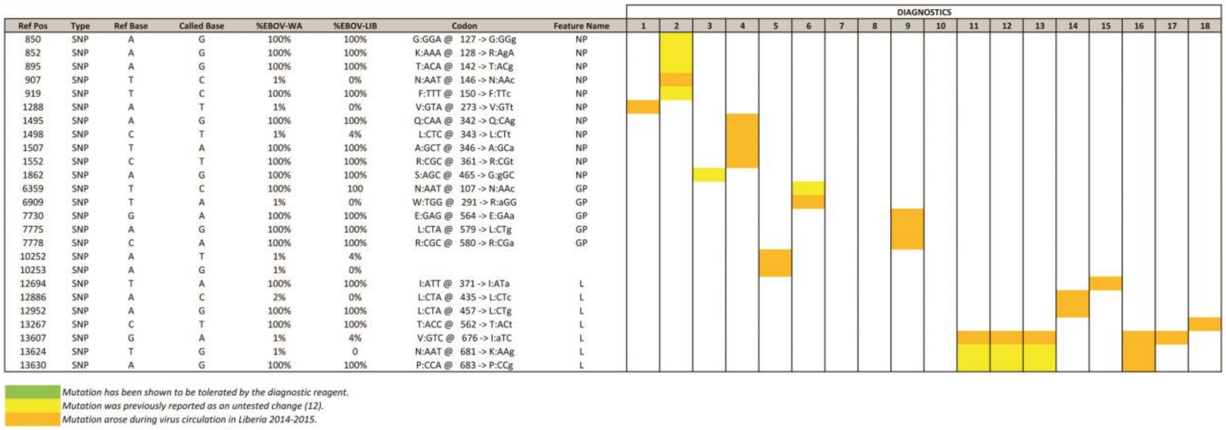

Ref Pos	Type	Ref Base	Called Base	%EBOV-WA	%EBOV-LIB	Codon	Feature Name	DIAGNOSTICS 1	2	3	4	5	6	7	8	9	10	11	12	13	14	15	16	17	18
850	SNP	A	G	100%	100%	G:GGA @ 127 -> G:GGg	NP																		
852	SNP	A	G	100%	100%	K:AAA @ 128 -> R:AgA	NP																		
895	SNP	A	G	100%	100%	T:ACA @ 142 -> T:ACg	NP																		
907	SNP	T	C	1%	0%	N:AAT @ 146 -> N:AAc	NP																		
919	SNP	T	C	100%	100%	F:TTT @ 150 -> F:TTc	NP																		
1288	SNP	A	T	1%	0%	V:GTA @ 273 -> V:GTt	NP																		
1495	SNP	A	G	100%	100%	Q:CAA @ 342 -> Q:CAg	NP																		
1498	SNP	C	T	1%	4%	L:CTC @ 343 -> L:CTt	NP																		
1507	SNP	T	A	100%	100%	A:GCT @ 346 -> A:GCa	NP																		
1552	SNP	C	T	100%	100%	R:CGC @ 361 -> R:CGt	NP																		
1862	SNP	A	G	100%	100%	S:AGC @ 465 -> G:gGC	NP																		
6359	SNP	T	C	100%	100	N:AAT @ 107 -> N:AAc	GP																		
6909	SNP	T	A	1%	0%	W:TGG @ 291 -> R:aGG	GP																		
7730	SNP	G	A	100%	100%	E:GAG @ 564 -> E:GAa	GP																		
7775	SNP	A	G	100%	100%	L:CTA @ 579 -> L:CTg	GP																		
7778	SNP	C	A	100%	100%	R:CGC @ 580 -> R:CGa	GP																		
10252	SNP	A	T	1%	4%																				
10253	SNP	A	G	1%	0%																				
12694	SNP	T	A	100%	100%	I:ATT @ 371 -> I:ATa	L																		
12886	SNP	A	C	2%	0%	L:CTA @ 435 -> L:CTc	L																		
12952	SNP	A	G	100%	100%	L:CTA @ 457 -> L:CTg	L																		
13267	SNP	C	T	100%	100%	T:ACC @ 562 -> T:ACt	L																		
13607	SNP	G	A	1%	4%	V:GTC @ 676 -> I:aTC	L																		
13624	SNP	T	G	1%	0	N:AAT @ 681 -> K:AAg	L																		
13630	SNP	A	G	100%	100%	P:CCA @ 683 -> P:CCg	L																		

Technical Appendix 1 Figure 2. Mutation analysis of diagnostic binding sites. A single-nucleotide polymorphism (SNP) table is combined with a heat map based on 2 categories: 1) mutations shown to be tolerated by the therapeutic or diagnostic target (highlighted in green); 2) mutations within the binding region of a therapeutic or diagnostic assay that have not yet been tested (highlighted in yellow/orange) (*20*–*24*,*27*,*30*,*31*); The column designated "% EBOV-WA" stratifies changes by the percentage of Western African sequences that support each mutation in comparison with EBOV/Kik-9510621. The column designated "% EBOV-LIB" stratifies changes by the percentage of Liberian sequences that support each mutation. Changes previously described are highlighted in yellow; changes that appeared during circulation in Liberia are highlighted in orange. The reference nucleotide positions reported here are in relation to EBOV/Kik (GenBank accession no. AY354458), which is one of the primary isolates used as reference for developing these therapeutic drugs and diagnostic assays. The changes to the probes are summarized in the Technical Appendix 1 Table.

Technical Appendix 1.Diagnostic probe information, used for Figure 2 and Supplementary Figure S4, highlighting mutated probe positions.

Figure references	Author	Gene	Name	Forward	Reverse	Probe	Reference
Fig. 2: Kulesh-MGB	Trombley, A	NP	Kulesh-MGB	TCTGACATGGATTACCACAAGATC	GGATGACTCTTTGCCGAACAA*T*C	AGGTCTGTCCGTTCAA	Am J Trop Med Hyg. 2010 May;82(5):954- 60. doi: 10.4269/ajtmh.2010.09-0636.
Fig. 2: Kulesh-TM	Trombley, A	GP	Kulesh-TM	TTTTCAATCCTCAACCGTAAGGC	CAGTCCGGTCCCAGAATGTG	GCAGCGATGGGG*C*GGCACATG	Am J Trop Med Hyg. 2010 May;82(5):954-60. doi: 10.4269/ajtmh.2010.09-0636.
Fig. S4 #1	Sanchez, A	NP	ZAI-NP	GGACCGCCAAGGT*A*AAAAATGA	GCATATTGTTGGAGTTGCTTCTCAGC		J Infect Dis. 1999 Feb;179 Suppl 1:S164- 9.
Fig. S4 #2	Towner, J.	NP	EboZNP	TGG*A*A*A*AAACATTAAGAGAACACTTGC	AGGAGAG*A*AACTGACCGGC*A* T	CATGCCGGAAGAGGAGAC*A*ACTGAAGC	Biosecur. Bioterror. 9, 361–371 (2011)
Fig. S4 #3	Weidmann, M.	NP	ENZ	ATGATGGA*A*GCTACGGCG	AGGACCAAGTCATCTGGTGC		J. Clin. Virol. 30, 94–99 (2004).
Fig. S4 #4	Trombley, A	NP	Ebola MGB-EBOV	*A*CT*C*AGAGAGGC*T*GCCACTG	CAAGTCCAAGATGGTCAAGTTC*G*		Am J Trop Med Hyg. 2010 May;82(5):954- 60. doi: 10.4269/ajtmh.2010.09-0636.
Fig. S4 #5	Gire, SK	VP24*	KGH	GTCGTTCC*AA*CAATCGAGCG	CGTCCCGTAGCTTTRGCCAT		Science. 2014 Sep 12;345(6202):1369- 72. doi: 10.1126/science.1259657. Epub 2014 Aug 28.
Fig. S4 #6	Sanchez, A	GP	EBO-GP	AATGGGCTGAAAA*T*TGCTACAATC	TTTTTTTAGTTTCCC*A*GAAGGCCCACT		J Infect Dis. 1999 Feb;179 Suppl 1:S164- 9.
Fig. S4 #7	Gunther, S.	GP	EBOGP	TGGGCTGAAAAYTGCTACAATC	CTTTGTGMACATASCGGCAC	CTACCAGCAGCGCCAGACGG	Antiviral Res. 2004 Sep;63(3):209-15.
Fig. S4 #8	Morvan, J. M.	GP	EBO1/2	TGGGTAATYATCCTYTTCCA	ACGACACCTTCAGCRAAAGT		Microbes Infect. 1, 1193–1201 (1999).
Fig. S4 #9	Strong, J.E.	GP	ZebovGP	GGCCAACGA*G*ACGACTCAA	AAAGGT*G*CG*T*AGCTCAGTTGTG		Proc Natl Acad Sci U S A. 2008 Nov 18;105(46):17982- 7. doi: 10.1073/pnas.0809698105. Epub 2008 Nov 3.
Fig. S4 #10	Morvan, J. M.	GP	EBO3/4	GTTTGTCGKGACAAACTGTC	TGGAARGCWAAGTCWCCGG		Microbes Infect. 1, 1193–1201 (1999).
Fig. S4 #11	Leroy, E	L	modified Filo AB	ATCGGAATTTTCTTTCTCATTGAAAGA	ATG*T*GGTGG*G*TTATAATAATCACTGA*C*ATGCAT		J Med Virol. 2000 Apr;60(4):463- 7.
Fig. S4 #12	Sanchez, A	L	Filo AB	ATCGGAATTTTCTTTCTCATT*	ATG*T*GGTGG*G*TTATAATAATCACTGA*C*ATG		J Infect Dis. 1999 Feb;179 Suppl 1:S164- 9.
Fig. S4 #13	Panning, M	L		AAGCATTTCCTAGCAATATGATGGT	ATG*T*GGTGG*G*TTATAATAATCACTGA*C*ATG	CC*A*AAATCATCACTIGTGTGGTGCCA	J Infect Dis. 2007 Nov 15;196 Suppl 2:S199-204.
Fig. S4 #14	Palacios, G.	L		*A*ACACCGGGTCTTAATTCTTATATCAA	GGTGGTAAAATTCCCATAG*T*AGTTCTTT		Emerg Infect Dis. 2006 Apr;12(4):692- 5.
Fig. S4 #15	Zhai, J.	L		TATTTTCCAT*T*CAAAAACACTGGG	GCTTCACAAAGTGTTTGAACATT		J Clin Microbiol. 2007 Jan;45(1):224- 6. Epub 2006 Nov 1.
Fig. S4 #16	Grard, G.	L	PanFilo-L1/2	ATMGRAAYTTTTCYTTYTCWYT	TG*W*GGHGGRYTATAAWARTCACTDA*C*AT		J. Infect. Dis. 204 (suppl. 3), S776–S784 (2011).

Figure references	Author	Gene	Name	Forward	Reverse	Probe	Reference
Fig. S4 #17	Grard, G.	L	PanFilo-L3/4	**GCNAARGCMTTYCCHAGYAAYATGATGG**	**ATAAWARTCACTDA*C*ATGCATRTARCA**		J. Infect. Dis. 204 (suppl. 3), S776–S784 (2011).
Fig. S4 #18		L	GAB-1	**GAATGTAGGTAGAAC*C*TTCGG**	**GCATATAACACTGTGGGATTG**		
